# Pre-operative antiplatelet therapy is associated with increased risk of periprosthetic joint infection following total shoulder arthroplasty

**DOI:** 10.1016/j.jsea.2026.100010

**Published:** 2026-03-03

**Authors:** Muhammad Hamza Ilyas, Mohammad Daher, Viraj Deshpande, Adam Lindsay, Joseph A. Abboud, Hafiz F. Kassam

**Affiliations:** a Massachusetts General Hospital, Boston, MA, USA; b Rothman Institute/Thomas Jefferson Medical Center, Philadelphia, PA, USA; c Sidney Kimmel Medical College, Thomas Jefferson University, Philadelphia, PA, USA; d St. Charles Center for Orthopedics and Neurosurgery, Bend, OR, USA; e Division of Shoulder and Elbow Surgery, Rothman Orthopaedic Institute, Philadelphia, PA, USA; f Department of Surgery, Hoag Orthopedics Institute, Irvine, CA, USA

**Keywords:** Total shoulder arthroplasty, Periprosthetic joint infection, Antiplatelet therapy, Aspirin, Clopidogrel, Postoperative complications

## Abstract

**Background:**

Antiplatelet therapy is widely prescribed for the prevention and management of cardiovascular disease and for mitigation of thromboembolic risk. However, its impact on post-operative periprosthetic joint infection (PJI) following total shoulder arthroplasty (TSA) remains poorly defined.

**Methods:**

A retrospective cohort study was conducted using the TriNetX US Research Network. Propensity score matching was performed to balance demographics, body mass index, and Charlson Comorbidity Index variables. The primary outcome was the incidence of PJI at 90 days, 6 months, and 1 year. Secondary outcomes included post-operative medical and surgical complications. Comparative risk was quantified using risk ratios with 95% confidence intervals. Subgroup analyses evaluated obese, frail, as well as antiplatelet subclass groups.

**Results:**

Following 1:1 propensity score matching, 51,020 patients remained in each cohort. Pre-operative antiplatelet use was associated with a significantly higher risk of PJI at 90 days (risk ratio [RR], 2.00), 6 months (RR, 1.87), and 1 year (RR, 1.80) (all *P* < .001). Secondary outcomes demonstrated higher rates of healthcare utilization, myocardial infarction, surgical site infection, and revision across all time points, while venous thromboembolism rates were largely comparable between groups. Subgroup analyses in obese and frail patients demonstrated persistently elevated risks associated with antiplatelet therapy. Dual antiplatelet therapy was associated with the highest complication rates, whereas low-dose aspirin demonstrated a more favorable safety profile compared with clopidogrel and high-dose aspirin.

**Conclusion:**

Pre-operative antiplatelet therapy in patients undergoing TSA was associated with an increased risk of PJI and higher rates of post-operative medical and surgical complications. These findings suggest that antiplatelet exposure may represent an important modifiable risk factor for infection following TSA and should be carefully considered during perioperative risk stratification and surgical optimization.

With the increasing life expectancy and the aging population, the total shoulder arthroplasty (TSA) is projected to reach an annual volume of 10 million cases by 2060.^6^ Although outpatient TSA is becoming increasingly common, non-orthopedic complications may still occur.^7^ Some of these major complications include cardiac events, stroke, and venous thromboembolism.^28^ To help mitigate these events, many surgeons implement the use of perioperative antiplatelet agents. In addition, some of the older patients requiring TSA may be on these drugs for their medical conditions, and while the drugs may be stopped pre-operatively, they are typically reinitiated early in the post-operative period.^25^

Antiplatelet agents may increase bleeding risk and may lead to complications such as hematoma and wound drainage.^3^^,^^17^^,^^25^ Hematomas are an iron-rich, low pH environment that reduces antibiotic penetrance and is more favorable to *Cutibacterium acnes* and staphylococcal growth and biofilm formation, increasing risk of wound dehiscence and ultimately providing a retrograde pathway for pathogens.^1^^,^^21^^,^^23^ Antiplatelet therapy induces early biochemical alterations that reduce platelet microbicidal activity and impair platelet–neutrophil signaling.^34^ Platelet dysfunction indirectly increases infection risk by affecting platelet-mediated innate immune response, altering COX-1 and COX-2 mediated cellular response, and inhibiting the synergistic effect of early leukocyte/platelet bacterial defense. Collectively, these mechanisms suggest a possible link between antiplatelet exposure and post-operative infection risk following TSA. However, whether antiplatelet agents directly increase the risk of periprosthetic joint infection (PJI) in shoulder arthroplasty is still not confirmed, and studies are reporting contradictory results.^2^^,^^19^^,^^20^

In TSA, limited data exist directly linking the use of anticoagulants and increases in risk of PJI post-operatively.^5^ Furthermore, no recent studies have examined the risk of PJI when using antiplatelets perioperatively. The aim of the present study is to determine the association between pre-operative use of anti-platelet therapy and PJI in TSA, utilizing a large national database.

## Methods

### Data source

This retrospective cohort study utilized data aggregated from the United States TriNetX Collaborative: a large, federated clinical research platform that captures deidentified electronic health record data from healthcare organizations across the United States. This network includes records from more than 120 million patients contributed by over 70 healthcare organizations. Data within TriNetX are harmonized using a standardized quality assurance framework that evaluates conformance, completeness, and plausibility to ensure analytic validity.^16^ The platform has been previously used to support high-quality observational research across multiple clinical domains.^29^ TriNetX operates in compliance with the Health Insurance Portability and Accountability Act and the General Data Protection Regulation. This study was reported in accordance with the Strengthening the Reporting of Observational Studies in Epidemiology guidelines.^10^ Institutional review board approval and informed consent were exempt because the study utilized deidentified data available through the TriNetX platform.

### Study participants

Patients aged 18 or older undergoing primary TSA were identified using the TriNetX Research Network based on Current Procedural Terminology code 23472. Pre-operative antiplatelet exposure was defined as any documented prescription for an antiplatelet agent within the 90 days preceding surgery. Patients were classified as antiplatelet users if they had at least one antiplatelet prescription within this 90-day window. Patients were excluded if they did not have a minimum of 1 year of post-operative follow-up. Baseline demographic characteristics and comorbidities were ascertained using International Classification of Diseases, 10th Revision, Clinical Modification (ICD-10-CM) diagnosis codes and Current Procedural Terminology codes. Patients were subsequently categorized according to their 5-factor modified frailty index (mFI-5) scores. Frailty was assessed using the mFI-5, a validated tool derived from the original 11-factor index developed through the American College of Surgeons National Surgical Quality Improvement Program.^18^^,^^24^^,^^32^ mFI-5 retained strong predictive validity by focusing on 5 routinely documented comorbidities: nonindependent functional status, hypertension, chronic obstructive pulmonary disease, congestive heart failure, and diabetes mellitus.^15^ In the present study, frailty components were defined using ICD-10-CM codes for essential hypertension (I10), problems related to care provider dependency (Z74), diabetes mellitus (E08–E13), chronic obstructive pulmonary disease (J44), and heart failure (I50).^31^ Obesity was defined as a body mass index greater than 30 kg/m^2^. Prespecified subgroup analyses were then performed with stratification by obesity status, severe frailty (mFI-5 ≥3), and antiplatelet subclasses to evaluate differential associations between antiplatelet exposure and post-operative outcomes.

### Outcomes and covariates

The primary outcome of interest was PJI following TSA, identified using ICD-10-CM diagnosis codes. Secondary outcomes included healthcare utilization measures (hospital readmission and emergency department visits), cardiovascular events (myocardial infarction), thromboembolic events (pulmonary embolism and deep vein thrombosis), surgical site infection, and revision shoulder arthroplasty. Outcomes were evaluated at 90 days, 6 months, and 1 year following the index procedure. Covariates were selected to account for potential confounding and included demographic variables (age, sex, and race), body mass index, tobacco and alcohol use, and comorbid conditions incorporated within the Charlson Comorbidity Index.

### 1:1 propensity score matching

Prior to matching, patients receiving antiplatelet therapy were older and demonstrated a higher prevalence of medical comorbidities, including diabetes mellitus, chronic kidney disease, cardiovascular disease, and cerebrovascular disease, compared with patients not receiving antiplatelet therapy. To address these baseline differences, 1:1 propensity score matching (PSM) was performed. PSM was performed using a caliper width of 0.1 pooled standard deviations of the 2 groups. Covariate balance between cohorts before and after matching was assessed using standardized mean differences, with an standardized mean difference less than 0.10 considered indicative of adequate balance. Following matching, 51,020 patients remained in each cohort (Table I).

**Table I:** Baseline demographic characteristics and comorbidities of patients undergoing primary total shoulder arthroplasty, before and after 1:1 propensity score matching, stratified by pre-operative antiplatelet therapy use.

Variable	Antiplatelet therapy cohort (N = 54,094)	No antiplatelet therapy cohort (N = 79,941)	*P* value	Antiplatelet therapy cohort (N = 51,020)	No antiplatelet therapy cohort (N = 51,020)	*P* value
Age at index (yr)	69.7 ± 9.9	68.0 ± 11.5	<.001	69.4 ± 9.9	69.4 ± 10.3	.848
Male	45.3%	43.5%	<.001	44.8%	44.8%	.995
Female	54.6%	56.5%	<.001	55.1%	55.1%	.940
White	87.8%	86.0%	<.001	87.6%	88.2%	.004
Black or African American	7.4%	7.4%	.706	7.4%	7.1%	.083
Hispanic or Latino	2.5%	3.2%	<.001	2.6%	2.4%	.074
Not Hispanic or Latino	79.3%	74.9%	<.001	78.9%	80.2%	<.001
BMI (kg/m^2^)	30.7 ± 6.8	30.6 ± 6.9	.001	30.8 ± 6.8	30.7 ± 6.8	.166
Tobacco use	4.9%	3.3%	<.001	4.4%	4.1%	.014
Alcohol abuse	3.8%	2.8%	<.001	3.6%	3.3%	.025
Osteoporosis	13.3%	10.4%	<.001	12.7%	12.5%	.331
Comorbidities within the Charlson comorbidity index						
Diabetes mellitus	27.4%	19.1%	<.001	25.6%	24.9%	.017
Chronic kidney disease	15.1%	9.4%	<.001	13.3%	12.5%	<.001
Liver disease	10.1%	7.9%	<.001	9.6%	9.1%	.011
Hypertension	69.5%	53.0%	<.001	67.7%	67.8%	.625
Heart failure	13.2%	7.0%	<.001	10.6%	9.8%	<.001
Peripheral vascular disease	8.4%	4.6%	<.001	6.7%	6.3%	.006
Acute myocardial infarction	6.5%	2.5%	<.001	4.2%	3.9%	.012
Cerebrovascular disease	15.4%	8.7%	<.001	12.9%	12.1%	<.001
Dementia	2.3%	1.3%	<.001	1.9%	1.7%	.095
COPD	14.6%	9.3%	<.001	12.9%	12.3%	.004
Rheumatoid disease	6.1%	4.7%	<.001	5.7%	5.4%	.031
Peptic ulcer disease	1.9%	1.1%	<.001	1.6%	1.4%	.026
Hemiplegia	1.1%	0.5%	<.001	0.8%	0.7%	.065
Paraplegia	0.3%	0.2%	.056	0.3%	0.3%	.951
HIV	0.4%	0.3%	.017	0.4%	0.3%	.184
Any malignancy	2.1%	1.5%	<.001	2.0%	1.8%	.154

*SD*, standardized difference; *BMI*, body mass index; *COPD*, chronic obstructive pulmonary disease.

All values are expressed as mean ± SD or percentage unless otherwise indicated.

After matching, all standardized differences were less than 0.1, confirming adequate covariate balance.

### Statistical analysis

Statistical analyses were performed using the TriNetX analytics platform, which utilizes Java, R, and Python for statistical computing. Comparisons were conducted pairwise between cohorts, and relative risks with corresponding 95% confidence intervals were calculated for each outcome. Statistical significance was defined as a 2-sided *P* value less than .05.

## Results

### Primary outcome

Pre-operative antiplatelet therapy was associated with a significantly increased risk of PJI at all evaluated time points. At 90 days, PJI occurred in 2.6% of patients receiving antiplatelet therapy compared with 1.3% in the no-antiplatelet cohort (risk ratio [RR], 2.00; 95% confidence interval [CI], 1.82-2.19; *P* < .001). This elevated risk persisted at 6 months (3.1% vs. 1.7%; RR, 1.87; 95% CI, 1.72-2.03; *P* < .001) and at 1 year (3.8% vs. 2.1%; RR, 1.80; 95% CI, 1.67-1.93; *P* < .001), demonstrating a consistent association between antiplatelet exposure and increased infection risk following TSA (Table II, Table III, Table IV).

**Table II:** Ninety-day postoperative outcomes following primary total shoulder arthroplasty following 1:1 propensity score matching, stratified by preoperative antiplatelet therapy.

Outcome	Antiplatelet	No antiplatelet	RR [95% CI]	*P* value
Readmission	0.8%	0.5%	1.440 [1.230-1.686]	<.001
ED visit	3.5%	2.9%	1.183 [1.086-1.290]	<.001
PE	0.3%	0.3%	1.011 [0.810-1.262]	.923
DVT	0.5%	0.4%	1.156 [0.963-1.389]	.120
MI	0.5%	0.3%	1.726 [1.399-2.129]	<.001
SSI	0.3%	0.2%	1.306 [1.016-1.677]	.036
PJI	2.6%	1.3%	1.999 [1.824-2.190]	<.001
Revision arthroplasty	1.0%	0.6%	1.758 [1.522-2.030]	<.001

*ED*, emergency department; *PE*, pulmonary embolism; *DVT*, deep vein thrombosis; *MI*, myocardial infarction; *SSI*, surgical site infection; *PJI*, periprosthetic joint infection.

**Table III:** Six-month postoperative outcomes following primary total shoulder arthroplasty following 1:1 propensity score matching, stratified by preoperative antiplatelet therapy.

Outcome	Antiplatelet	No antiplatelet	RR [95% CI]	*P* value
Readmission	0.9%	0.7%	1.385 [1.201-1.599]	<.001
ED visit	5.3%	4.5%	1.170 [1.092-1.253]	<.001
PE	0.4%	0.5%	0.957 [0.795-1.152]	.640
DVT	0.8%	0.6%	1.193 [1.026-1.387]	.022
MI	0.7%	0.5%	1.512 [1.281-1.784]	<.001
SSI	0.4%	0.3%	1.348 [1.079-1.684]	.008
PJI	3.1%	1.7%	1.869 [1.720-2.030]	<.001
Revision arthroplasty	1.5%	0.9%	1.710 [1.520-1.925]	<.001

*ED*, emergency department; *PE*, pulmonary embolism; *DVT*, deep vein thrombosis; *MI*, myocardial infarction; *SSI*, surgical site infection; *PJI*, periprosthetic joint infection.

**Table IV:** One-year postoperative outcomes following primary total shoulder arthroplasty following 1:1 propensity score matching, stratified by preoperative antiplatelet therapy.

Outcome	Antiplatelet	No antiplatelet	RR [95% CI]	*P* value
Readmission	1.2%	0.8%	1.418 [1.248-1.612]	<.001
ED visit	8.5%	7.7%	1.104 [1.048-1.164]	<.001
PE	0.7%	0.7%	1.044 [0.901-1.210]	.568
DVT	1.1%	0.9%	1.160 [1.025-1.313]	.018
MI	1.3%	0.8%	1.528 [1.350-1.729]	<.001
SSI	0.5%	0.3%	1.540 [1.265-1.875]	<.001
PJI	3.8%	2.1%	1.795 [1.667-1.932]	<.001
Revision arthroplasty	2.1%	1.2%	1.718 [1.555-1.898]	<.001

*ED*, emergency department; *PE*, pulmonary embolism; *DVT*, deep vein thrombosis; *MI*, myocardial infarction; *SSI*, surgical site infection; *PJI*, periprosthetic joint infection.

### Secondary outcomes

#### Healthcare utilization

Readmission rates increased at 90 days (0.8% vs. 0.5%; RR, 1.44; *P* < .001), 6 months (0.9% vs. 0.7%; RR, 1.39; *P* < .001), and 1 year (1.2% vs. 0.8%; RR, 1.42; *P* < .001). Emergency department utilization was similarly higher among antiplatelet users at 90 days (3.5% vs. 2.9%; RR, 1.18; *P* < .001), 6 months (5.3% vs. 4.5%; RR, 1.17; *P* < .001), and 1 year (8.5% vs. 7.7%; RR, 1.10; *P* < .001) (Table II, Table III, Table IV).

#### Cardiovascular and thromboembolic events

Myocardial infarction occurred more frequently among patients receiving antiplatelet therapy at all time points, including 90 days (0.5% vs. 0.3%; RR, 1.73; *P* < .001), 6 months (0.7% vs. 0.5%; RR, 1.51; *P* < .001), and 1 year (1.3% vs. 0.8%; RR, 1.53; *P* < .001). In contrast, rates of pulmonary embolism were comparable between cohorts at 90 days, 6 months, and 1 year (*P* > .05 for all comparisons). Deep vein thrombosis rates were similar at 90 days but were modestly higher among antiplatelet users at 6 months (0.8% vs. 0.6%; RR, 1.19; *P* = .022) and at 1 year (1.1% vs. 0.9%; RR, 1.16; *P* = .018) (Table II, Table III, Table IV).

#### Surgical complications and revision

Surgical site infection occurred more frequently in the antiplatelet cohort at 90 days (0.3% vs. 0.2%; RR, 1.31; *P* = .036), 6 months (0.4% vs. 0.3%; RR, 1.35; *P* = .008), and 1 year (0.5% vs. 0.3%; RR, 1.54; *P* < .001). Revision arthroplasty rates were also significantly higher among patients receiving antiplatelet therapy at 90 days (1.0% vs. 0.6%; RR, 1.76; *P* < .001), 6 months (1.5% vs. 0.9%; RR, 1.71; *P* < .001), and 1 year (2.1% vs. 1.2%; RR, 1.72; *P* < .001) (Table II, Table III, Table IV).

### Subgroup analysis

Across subgroup and medication-specific analyses, a graded risk hierarchy was observed. The lowest overall complication and utilization risk was seen with aspirin 81 mg, followed by aspirin 325 mg, clopidogrel, and the highest risk with dual antiplatelet therapy.

#### Obese patients (body mass index >30 kg/m^2^)

Among obese patients, pre-operative antiplatelet use was associated with increased risks of readmission (RR, 1.31), emergency department utilization (RR, 1.30), myocardial infarction (RR, 2.73), PJI (RR, 2.03), and revision arthroplasty (RR, 1.99) at 90 days. These associations persisted through 6 months and 1 year, with antiplatelet users demonstrating approximately twofold higher risks of myocardial infarction (RR range, 2.00-2.29), PJI (RR range, 1.85-1.94), and revision surgery (RR range, 1.99-2.04), while pulmonary embolism and deep vein thrombosis rates were similar between groups (Table V, Table VI, Table VII).

**Table V:** Ninety-day postoperative outcomes following primary total shoulder arthroplasty after propensity score matching in obese patients.

Outcome	Antiplatelet (n = 26,915)	No antiplatelet (n = 26,915)	RR [95% CI]	*P* value
Readmission	1.3%	1.0%	1.313 [1.121, 1.538]	**.001**
ED visit	12.6%	9.7%	1.302 [1.241, 1.366]	**<.001**
PE	1.4%	1.3%	1.096 [0.948, 1.268]	.215
DVT	1.4%	1.6%	0.913 [0.796, 1.046]	.190
MI	2.5%	0.9%	2.727 [2.358, 3.153]	<.001
SSI	0.3%	0.3%	1.184 [0.873, 1.606]	.276
PJI	3.1%	1.6%	2.026 [1.805, 2.275]	**<.001**
Revision arthroplasty	1.3%	0.7%	1.994 [1.665, 2.389]	**<.001**

*ED*, emergency department; *PE*, pulmonary embolism; *DVT*, deep vein thrombosis; *MI*, myocardial infarction; *SSI*, surgical site infection; *PJI*, periprosthetic joint infection.

Bold values indicate statistical significance (*P* < .05).

**Table VI:** Six months postoperative outcomes following primary total shoulder arthroplasty after propensity score matching in obese patients.

Outcome	Antiplatelet (n = 26,915)	No antiplatelet (n = 26,915)	RR [95% CI]	*P* value
Readmission	1.5%	1.2%	1.337 [1.155, 1.546]	**<.001**
ED visit	18.0%	14.3%	1.258 [1.210, 1.307]	**<.001**
PE	1.7%	1.6%	1.041 [0.914, 1.185]	.545
DVT	1.9%	2.0%	0.927 [0.822, 1.045]	.213
MI	3.1%	2.0%	2.288 [2.026, 2.585]	**<.001**
SSI	0.4%	0.4%	1.221 [0.931, 1.601]	.147
PJI	3.7%	1.9%	1.934 [1.741, 2.148]	**<.001**
Revision arthroplasty	1.9%	0.9%	2.044 [1.759, 2.374]	**<.001**

*ED*, emergency department; *PE*, pulmonary embolism; *DVT*, deep vein thrombosis; *MI*, myocardial infarction; *SSI*, surgical site infection; *PJI*, periprosthetic joint infection.

Bold values indicate statistical significance (*P* < .05).

**Table VII:** One-year postoperative outcomes following primary total shoulder arthroplasty after propensity score matching in obese patients.

Outcome	Antiplatelet (n = 26,915)	No antiplatelet (n = 26,915)	RR [95% CI]	*P* value
Readmission	2.0%	1.4%	1.431 [1.253, 1.633]	**<.001**
ED visit	25.6%	21.1%	1.212 [1.176, 1.250]	**<.001**
PE	2.2%	2.2%	1.003 [0.897, 1.122]	.953
DVT	2.5%	2.7%	0.933 [0.842, 1.034]	.187
MI	4.1%	2.0%	2.000 [1.808, 2.212]	**<.001**
SSI	0.6%	0.5%	1.358 [1.076, 1.713]	.010
PJI	4.4%	2.4%	1.852 [1.684, 2.035]	**<.001**
Revision arthroplasty	2.6%	1.3%	1.991 [1.752, 2.263]	**<.001**

*ED*, emergency department; *PE*, pulmonary embolism; *DVT*, deep vein thrombosis; *MI*, myocardial infarction; *SSI*, surgical site infection; *PJI*, periprosthetic joint infection.

Bold values indicate statistical significance (*P* < .05).

#### Frail patients (modified frailty index ≥3)

At 90 days, antiplatelet users demonstrated increased risks of myocardial infarction (0.5% vs. 0.3%; RR, 1.89; *P* < .001), PJI (0.9% vs. 0.6%; RR, 1.56; *P* < .001), and revision arthroplasty (0.9% vs. 0.5%; RR, 1.87; *P* < .001). At 6 months and 1 year, risks of myocardial infarction and revision remained significantly elevated, while differences in PJI were attenuated and did not reach statistical significance. Thromboembolic event rates were similar between cohorts at all time points (Table VIII, Table IX, Table X).

**Table VIII:** Ninety-day postoperative outcomes following primary total shoulder arthroplasty in a propensity-matched subgroup analysis (mFI ≥ 3).

Outcome	Antiplatelet (n = 5,601)	No antiplatelet (n = 5,601)	RR [95% CI]	*P* value
Readmission	0.7%	0.5%	1.355 [1.132, 1.623]	**.001**
ED visit	3.5%	3.0%	1.149 [1.043, 1.266]	**.005**
PE	0.3%	0.3%	1.054 [0.826, 1.345]	.674
DVT	0.5%	0.5%	1.050 [0.859, 1.282]	.635
MI	0.5%	0.3%	1.887 [1.494, 2.383]	**<.001**
SSI	0.2%	0.2%	1.184 [0.874, 1.603]	.275
PJI	0.9%	0.6%	1.558 [1.316, 1.844]	**<.001**
Revision arthroplasty	0.9%	0.5%	1.866 [1.573, 2.215]	**<.001**

*ED*, emergency department; *PE*, pulmonary embolism; *DVT*, deep vein thrombosis; *MI*, myocardial infarction; *SSI*, surgical site infection; *PJI*, periprosthetic joint infection; *mFI*, modified frailty index.

Bold values indicate statistical significance (*P* < .05).

**Table IX:** Six months postoperative outcomes following primary total shoulder arthroplasty in severe frail patients (mFI-5 ≥ 3).

Outcome	Antiplatelet (n = 5,601)	No antiplatelet (n = 5,601)	RR [95% CI]	*P* value
Readmission	1.5%	0.9%	1.619 [1.130, 2.319]	**.008**
ED visit	10.2%	7.7%	1.329 [1.076, 1.642]	**.008**
PE	0.8%	0.9%	0.809 [0.534, 1.226]	.317
DVT	1.4%	1.3%	1.125 [0.806, 1.571]	.487
MI	2.5%	1.6%	1.541 [1.155, 2.057]	**.003**
SSI	0.7%	0.5%	1.524 [0.921, 2.521]	.098
PJI	1.2%	1.1%	1.154 [0.807, 1.651]	.432
Revision arthroplasty	1.1%	0.6%	1.842 [1.213, 2.797]	**.004**

*ED*, emergency department; *PE*, pulmonary embolism; *DVT*, deep vein thrombosis; *MI*, myocardial infarction; *SSI*, surgical site infection; *PJI*, periprosthetic joint infection; *mFI*, modified frailty index.

Bold values indicate statistical significance (*P* < .05).

**Table X:** One-year postoperative outcomes following primary total shoulder arthroplasty in severe frail patients (mFI-5 ≥3).

Outcome	Antiplatelet (n = 5,601)	No antiplatelet (n = 5,601)	RR [95% CI]	*P* value
Readmission	1.8%	1.1%	1.662 [1.194, 2.313]	**.002**
ED visit	14.8%	11.5%	1.281 [1.081, 1.519]	**.004**
PE	1.4%	1.5%	0.927 [0.674, 1.275]	.641
DVT	2.3%	1.9%	1.209 [0.922, 1.586]	.169
MI	4.4%	2.8%	1.584 [1.274, 1.970]	**<.001**
SSI	0.9%	0.7%	1.409 [0.926, 2.144]	.108
PJI	1.7%	1.4%	1.188 [0.876, 1.611]	.267
Revision arthroplasty	1.7%	1.0%	1.661 [1.189, 2.320]	**.003**

*ED*, emergency department; *PE*, pulmonary embolism; *DVT*, deep vein thrombosis; *MI*, myocardial infarction; *SSI*, surgical site infection; *PJI*, periprosthetic joint infection; *mFI*, modified frailty index.

Bold values indicate statistical significance (*P* < .05).

#### Dual antiplatelet therapy vs. no antiplatelet therapy

Dual antiplatelet therapy was associated with the highest complication burden among all medication subgroups. At 90 days, combination therapy was associated with increased risks of myocardial infarction (RR, 1.89), PJI (RR, 1.56), and revision arthroplasty (RR, 1.87) compared with no antiplatelet use (all *P* ≤ .001). These associations persisted for 6 months and 1 year. At 1 year, dual antiplatelet therapy was associated with increased risks of myocardial infarction (1.5% vs. 1.3%; RR, 1.62), PJI (1.6% vs. 1.2%; RR, 1.42), and revision surgery (2.0% vs. 1.1%; RR, 1.81) (all *P* < .001). Rates of pulmonary embolism were similar between groups, while deep vein thrombosis was modestly increased at 1 year (Supplementary Tables S1-S3).

#### Aspirin 81 mg vs. clopidogrel

Aspirin 81 mg was associated with significantly lower readmission rates at 90 days (0.6% vs. 1.3%), 180 days (0.7% vs. 1.4%), and 1 year (0.9% vs. 1.7%) compared with clopidogrel (all *P* < .001). Rates of all other complications were comparable between groups at all follow-up intervals. Overall, aspirin 81 mg demonstrated a more favorable utilization profile without differences in thrombotic, infectious, or reoperation outcomes relative to clopidogrel (Supplementary Tables S4-S6).

#### Aspirin 81 mg vs. aspirin 325 mg

Aspirin 81 mg demonstrated the lowest thrombotic risk, with consistently lower rates of deep vein thrombosis at 90 days (0.4% vs. 0.6%), 180 days (0.7% vs. 0.9%), and 1 year (1.1% vs. 1.3%) compared with aspirin 325 mg, without differences in pulmonary embolism or myocardial infarction. Although low-dose aspirin was associated with statistically higher rates of surgical site infection and revision, the absolute differences were small and were not accompanied by any difference in PJI rates at 90 days, 6 months, or 1 year (Supplementary Tables S7-S9).

## Discussion

Our study demonstrated that antiplatelet use was associated with nearly doubled rates of PJI and revision arthroplasty at 90 days, 6 months, and 1 year, along with a 1.5- to 2-fold increase in myocardial infarction and higher healthcare utilization across all time points. These patterns were consistent across subgroup analyses, including obese and frail patients, and were most pronounced among patients receiving dual antiplatelet regimens. In contrast, thromboembolic event rates were largely similar between cohorts, with pulmonary embolism remaining unchanged and deep vein thrombosis risk lowest among patients receiving low-dose aspirin. Low-dose aspirin approached a 20-30% relative reduction compared with higher-dose aspirin. Collectively, these findings indicate that perioperative antiplatelet exposure is associated with a substantially elevated post-operative risk profile following TSA, driven predominantly by infection and revision.

Arthroplasty-associated infection, or PJI, is an uncommon but clinically distinct condition compared with native bone or joint infection. PJI arises from complex interactions between invading microorganisms, the implanted prosthesis, and the host immune response. Even small quantities of microorganisms may result in infection, as bacteria and less common fungi can adhere to implant surfaces and form biofilms.^22^ Similarly, early disruptions in the leukocyte-platelet coordination may allow for a lower threshold for bacterial penetration. PJI is a leading cause of revision surgery, prolonged hospitalization, and functional decline, often necessitating complex reoperations and extended antimicrobial therapy.^26^

Shoulder PJI imposes a disproportionate financial burden driven by increased resource utilization, repeat procedures, and prolonged care pathways. From 2011 to 2018, total hospital charges attributable to shoulder PJI increased more than threefold, reaching nearly $200 million annually, with projections estimating an escalation to approximately $500 million in annual charges by 2030.^26^ The findings of this study reinforce that even modest elevations in the risk of PJI are clinically consequential, with important implications for patient outcomes and a substantial associated healthcare burden, highlighting the importance of identifying perioperative risk factors that may be amenable to targeted optimization.

Platelets play an important role in host defense through several mechanisms that may be disrupted by antiplatelet therapy. Activated platelets release antimicrobial peptides from α-granules that directly inhibit bacterial growth, particularly against *Staphylococcus aureus*, the most common pathogen implicated in PJI.^13^^,^^14^ In addition, platelet activation induces surface expression of P-selectin, facilitating recruitment and activation of neutrophils and monocytes and promoting the release of cytokines and proteolytic enzymes that enhance innate immune responses. P2Y12 inhibition—directly affected by P2Y12 inhibitors (clopidogrel)—reduces platelet–neutrophil aggregates, decreasing neutrophil migration, phagocytosis, and oxidative burst at wounds. Platelets also interact directly with bacteria through multiple surface receptors, enabling bacterial binding and, in some contexts, internalization.^8^^,^^13^

Antiplatelet therapy has been shown to impair these antimicrobial functions. Hannachi et al demonstrated that aspirin, ticagrelor, and combination antiplatelet therapy significantly attenuated antibacterial activity, indicating that antiplatelet exposure impairs platelet dependent antimicrobial function.^14^ Schrottmaier et al demonstrated that inhibition of cyclooxygenase and P2Y12 signaling pathways with aspirin and P2Y12 inhibitors including clopidogrel markedly reduces platelet–leukocyte aggregate formation.^27^

Certainly, hematoma formation at the surgical site is directly linked to an increased risk of infection.^11^ Dual antiplatelet therapy, particularly aspirin combined with clopidogrel, significantly increases hematoma risk.^30^^,^^33^ Hematomas may cause tension leading to wound breach and post-operative contamination, pressure-induced tissue necrosis, and provide a favorable environment for bacterial colonization.^11^ Consistent with this mechanism, de Haan et al demonstrated that post-operative hematoma was a strong independent predictor of PJI following hip hemiarthroplasty, conferring more than a sixfold increased risk after multivariable adjustment.^12^ These findings suggest that antiplatelet therapy may be associated with increased susceptibility to PJI through a dual mechanism involving impaired platelet-dependent immune defense and increased bleeding-related wound complications, providing biologic context for the observed associations.

Evidence from prior clinical studies supports an association between the observed findings. Blasco-Colmenares et al reported higher rates of post-operative surgical site infection and bacteremia among patients receiving dual antiplatelet therapy compared with aspirin alone following coronary artery bypass surgery.^4^ Pai et al evaluated the association between venous thromboembolism prophylaxis and early post-operative infection following primary total joint arthroplasty and found that its use was associated with higher odds of 90-day readmission for surgical site complications (adjusted odds ratio [aOR], 1.75; 95% CI, 1.08-2.84), 90-day readmission for PJI (aOR, 3.27; 95% CI, 1.03-10.40), and overall 90-day PJI events (aOR, 3.22; 95% CI, 1.20-8.66).^20^ Similarly, Sumarriva et al observed a markedly higher rate of PJI among patients receiving both antiplatelet and anticoagulant therapy within 90 days of total joint arthroplasty, while rates of pulmonary embolism and deep vein thrombosis remained similar between groups.^28^

Future prospective studies are needed to evaluate the impact of antiplatelet agent class, doses, and duration of exposure, as well as the timing of perioperative interruption or continuation, on both infectious and thrombotic outcomes. These studies should take into consideration the current CHEST (American College of Chest Physicians) and American Heart Association/American College of Cardiology) pre-operative antiplatelet management guidelines, which recommend continuation of aspirin preoperatively and medication specific cessation of other antiplatelet therapy.^9^ In addition, risk-stratified clinical trials and comparative effectiveness studies are needed to determine whether individualized perioperative antiplatelet management strategies can mitigate infection risk, thereby optimizing outcomes for patients undergoing TSA.

This study has several limitations inherent to its retrospective, observational design. First, analyses were conducted using a large electronic health record database, which is subject to misclassification, undercoding, and overcoding of diagnoses and procedures, particularly with reliance on ICD-10 coding. As such, the true incidence of complications, including PJI, may be underestimated or misclassified. Second, granular data regarding antiplatelet indication, dosage, duration of therapy, adherence, perioperative discontinuation timing, and post-operative resumption were unavailable, limiting evaluation of dose–response relationships and duration-dependent effects. In particular, variability in whether antiplatelet agents were continued, temporarily withheld, or resumed early after surgery may influence outcomes but could not be accounted for in the present analysis. Third, confounding by indication remains likely, as patients prescribed antiplatelet therapy may have greater underlying medical complexity. In addition, lack of perioperative variables, including intraoperative blood loss, post-operative hematoma or wound drainage, and antibiotic prophylaxis protocols, limits assessment of potential mediators of infection risk. Fourth, the higher rates of myocardial infarction observed among patients receiving antiplatelet therapy may reflect greater underlying cardiovascular risk rather than a direct pharmacologic effect. Fifth, TriNetX is an opt-in database composed predominantly of large, research-oriented healthcare systems, which may limit generalizability to smaller or non-academic institutions. Finally, although PSM was performed to balance measured confounders, residual confounding from unmeasured variables remains possible and causal inferences cannot be established.

## Conclusion

In this large, propensity-matched analysis of patients undergoing TSA, pre-operative antiplatelet therapy was associated with a significantly increased risk of PJI and higher rates of post-operative medical and surgical complications. Complication rates varied by antiplatelet regimen, with the most favorable complication profile observed among patients receiving low-dose aspirin and progressively higher risks seen with dual antiplatelet therapy. Perioperative antiplatelet exposure may be associated with impaired host defense mechanisms and may represent an important, potentially modifiable risk factor for infection following TSA. Careful perioperative risk stratification and individualized antiplatelet management should be considered to balance thrombotic protection against infectious risk in this growing surgical population.

## Disclaimers

Funding: No funding was disclosed by the authors.

Conflicts of interest: Adam Lindsay is a consultant for Stryker. Joseph Abboud has received royalties from Osteocentric Technologies, Enovis, Zimmer Biomet, Stryker, and Globus Medical; has stocks in Shoulder JAM, Aevumed, OBERD, OTS Medical, Orthobullets, Atreon, and Restore 3D; has received research support from Enovis and Arthrex; has received royalties or financial or material support from Wolters Kluwer, Slack Orthopaedics, and Elsevier; and is a board member or has committee appointments for the American Shoulder and Elbow Society, Mid Atlantic Shoulder and Elbow Society, Shoulder 360, and Pacira. Hafiz Kassam is a consultant for Zimmer Biomet and Smith & Nephew. Any additional authors, their immediate families, and any research foundations with which they are affiliated have not received any financial payments or other benefits from any commercial entity related to the subject of this article.
