# Supplementary material for: Pre-operative antiplatelet therapy is associated with increased risk of periprosthetic joint infection following total shoulder arthroplasty
Source: J Shoulder Elb Arthroplast. 2026 Mar 3;10(1-2):100010. doi: 10.1016/j.jsea.2026.100010 (PMC13103263; doi:10.1016/j.jsea.2026.100010)
Supplement: Supplementary Table 1 [file mmc1.docx]

*Supplementary Table 1. Ninety-Day Postoperative Outcomes Following Primary Total Shoulder Arthroplasty in Patients Receiving Dual Antiplatelet Therapy (Aspirin + Clopidogrel) Compared with No Antiplatelet Therapy*

| Outcome | Aspirin + Clopidogrel (n = 40,954) | No Antiplatelet (n = 40,954) | RR [95% CI] | P value |
| --- | --- | --- | --- | --- |
| Readmission | 0.7% | 0.5% | 1.355 [1.132, 1.623] | **0.001** |
| ED Visit | 3.5% | 3.0% | 1.149 [1.043, 1.266] | **0.005** |
| PE | 0.3% | 0.3% | 1.054 [0.826, 1.345] | 0.674 |
| DVT | 0.5% | 0.5% | 1.050 [0.859, 1.282] | 0.635 |
| MI | 0.5% | 0.3% | 1.887 [1.494, 2.383] | **<0.001** |
| SSI | 0.2% | 0.2% | 1.184 [0.874, 1.603] | 0.275 |
| PJI | 0.9% | 0.6% | 1.558 [1.316, 1.844] | **<0.001** |
| Revision Arthroplasty | 0.9% | 0.5% | 1.866 [1.573, 2.215] | **<0.001** |
