# Supplementary material for: Pre-operative antiplatelet therapy is associated with increased risk of periprosthetic joint infection following total shoulder arthroplasty
Source: J Shoulder Elb Arthroplast. 2026 Mar 3;10(1-2):100010. doi: 10.1016/j.jsea.2026.100010 (PMC13103263; doi:10.1016/j.jsea.2026.100010)
Supplement: Supplementary Table 2 [file mmc2.docx]

*Supplementary Table 2. Six-Months Postoperative Outcomes Following Primary Total Shoulder Arthroplasty in Patients Receiving Dual Antiplatelet Therapy (Aspirin + Clopidogrel) Compared with No Antiplatelet Therapy*

| Outcome | Aspirin + Clopidogrel (n = 40,954) | No Antiplatelet (n = 40,954) | RR [95% CI] | P value |
| --- | --- | --- | --- | --- |
| Readmission | 0.8% | 0.6% | 1.340 [1.137, 1.579] | **<0.001** |
| ED Visit | 5.4% | 4.7% | 1.136 [1.052, 1.226] | **0.001** |
| PE | 0.5% | 0.5% | 1.022 [0.836, 1.251] | 0.829 |
| DVT | 0.7% | 0.7% | 1.084 [0.919, 1.279] | 0.340 |
| MI | 0.8% | 0.5% | 1.647 [1.377, 1.971] | **<0.001** |
| SSI | 0.3% | 0.2% | 1.295 [0.987, 1.700] | 0.061 |
| PJI | 1.2% | 0.8% | 1.444 [1.252, 1.665] | **<0.001** |
| Revision Arthroplasty | 1.4% | 0.7% | 1.859 [1.616, 2.139] | **<0.001** |
