# Supplementary material for: Pre-operative antiplatelet therapy is associated with increased risk of periprosthetic joint infection following total shoulder arthroplasty
Source: J Shoulder Elb Arthroplast. 2026 Mar 3;10(1-2):100010. doi: 10.1016/j.jsea.2026.100010 (PMC13103263; doi:10.1016/j.jsea.2026.100010)
Supplement: Supplementary Table 3 [file mmc3.docx]

*Supplementary Table 3. One-Year Postoperative Outcomes Following Primary Total Shoulder Arthroplasty in Patients Receiving Dual Antiplatelet Therapy (Aspirin + Clopidogrel) Compared with No Antiplatelet Therapy*

| Outcome | Aspirin + Clopidogrel (n = 40,954) | No Antiplatelet (n = 40,954) | RR [95% CI] | P value |
| --- | --- | --- | --- | --- |
| Readmission | 1.1% | 0.8% | 1.398 [1.207, 1.620] | **<0.001** |
| ED Visit | 8.7% | 7.9% | 1.101 [1.038, 1.167] | **0.001** |
| PE | 0.8% | 0.7% | 1.082 [0.921, 1.272] | 0.337 |
| DVT | 1.1% | 1.0% | 1.161 [1.013, 1.331] | 0.032 |
| MI | 1.5% | 1.3% | 1.617 [1.416, 1.847] | **<0.001** |
| SSI | 0.4% | 0.3% | 1.533 [1.212, 1.938] | **<0.001** |
| PJI | 1.6% | 1.2% | 1.420 [1.262, 1.599] | **<0.001** |
| Revision Arthroplasty | 2.0% | 1.1% | 1.810 [1.612, 2.031] | **<0.001** |
