# Supplementary material for: Pre-operative antiplatelet therapy is associated with increased risk of periprosthetic joint infection following total shoulder arthroplasty
Source: J Shoulder Elb Arthroplast. 2026 Mar 3;10(1-2):100010. doi: 10.1016/j.jsea.2026.100010 (PMC13103263; doi:10.1016/j.jsea.2026.100010)
Supplement: Supplementary Table 4 [file mmc4.docx]

*Supplementary Table 4. Ninety-Day Postoperative Outcomes Following Primary Total Shoulder Arthroplasty Comparing Low-Dose Aspirin (81 mg) and Clopidogrel*

| Outcome | Aspirin 81 mg (n = 5,910) | Clopidogrel (n = 5,910) | RR [95% CI] | P value |
| --- | --- | --- | --- | --- |
| Readmission | 0.6% | 1.3% | 0.461 [0.306, 0.696] | **<0.001** |
| ED Visit | 5.0% | 5.7% | 0.877 [0.690, 1.115] | 0.284 |
| PE | 0.6% | 0.5% | 1.141 [0.694, 1.877] | 0.603 |
| DVT | 0.7% | 0.6% | 1.079 [0.683, 1.705] | 0.746 |
| MI | 1.2% | 1.5% | 0.833 [0.584, 1.188] | 0.313 |
| SSI | 0.3% | 0.4% | 0.763 [0.399, 1.461] | 0.413 |
| PJI | 0.7% | 0.8% | 0.867 [0.568, 1.322] | 0.507 |
| Revision Arthroplasty | 1.0% | 1.0% | 1.015 [0.706, 1.461] | 0.935 |
