# Supplementary material for: Pre-operative antiplatelet therapy is associated with increased risk of periprosthetic joint infection following total shoulder arthroplasty
Source: J Shoulder Elb Arthroplast. 2026 Mar 3;10(1-2):100010. doi: 10.1016/j.jsea.2026.100010 (PMC13103263; doi:10.1016/j.jsea.2026.100010)
Supplement: Supplementary Table 5 [file mmc5.docx]

*Supplementary Table 5. Six-Months Postoperative Outcomes Following Primary Total Shoulder Arthroplasty Comparing Low-Dose Aspirin (81 mg) and Clopidogrel*

| Outcome | Aspirin 81 mg (n = 5,910) | Clopidogrel (n = 5,910) | RR [95% CI] | P value |
| --- | --- | --- | --- | --- |
| Readmission | 0.7% | 1.4% | 0.509 [0.348, 0.744] | **<0.001** |
| ED Visit | 7.6% | 8.4% | 0.903 [0.744, 1.096] | 0.300 |
| PE | 0.8% | 0.7% | 1.106 [0.718, 1.703] | 0.648 |
| DVT | 0.9% | 1.0% | 0.869 [0.594, 1.273] | 0.472 |
| MI | 1.8% | 2.2% | 0.815 [0.607, 1.093] | 0.171 |
| SSI | 0.4% | 0.5% | 0.853 [0.490, 1.486] | 0.575 |
| PJI | 0.9% | 1.1% | 0.810 [0.563, 1.166] | 0.256 |
| Revision Arthroplasty | 1.5% | 1.3% | 1.144 [0.841, 1.556] | 0.391 |
