# Supplementary material for: Pre-operative antiplatelet therapy is associated with increased risk of periprosthetic joint infection following total shoulder arthroplasty
Source: J Shoulder Elb Arthroplast. 2026 Mar 3;10(1-2):100010. doi: 10.1016/j.jsea.2026.100010 (PMC13103263; doi:10.1016/j.jsea.2026.100010)
Supplement: Supplementary Table 6 [file mmc6.docx]

*Supplementary Table 6. One-Year Postoperative Outcomes Following Primary Total Shoulder Arthroplasty Comparing Low-Dose Aspirin (81 mg) and Clopidogrel*

| Outcome | Aspirin 81 mg (n = 5,910) | Clopidogrel (n = 5,910) | RR [95% CI] | P value |
| --- | --- | --- | --- | --- |
| Readmission | 0.9% | 1.7% | 0.496 [0.350, 0.703] | **<0.001** |
| ED Visit | 11.7% | 12.4% | 0.942 [0.808, 1.099] | 0.446 |
| PE | 1.3% | 1.2% | 1.074 [0.777, 1.485] | 0.664 |
| DVT | 1.6% | 1.5% | 1.005 [0.747, 1.353] | 0.973 |
| MI | 3.1% | 3.8% | 0.817 [0.655, 1.018] | 0.071 |
| SSI | 0.6% | 0.7% | 0.899 [0.570, 1.417] | 0.646 |
| PJI | 1.5% | 1.6% | 0.899 [0.670, 1.207] | 0.480 |
| Revision Arthroplasty | 1.9% | 1.8% | 1.077 [0.823, 1.408] | 0.589 |
