# Supplementary material for: Pre-operative antiplatelet therapy is associated with increased risk of periprosthetic joint infection following total shoulder arthroplasty
Source: J Shoulder Elb Arthroplast. 2026 Mar 3;10(1-2):100010. doi: 10.1016/j.jsea.2026.100010 (PMC13103263; doi:10.1016/j.jsea.2026.100010)
Supplement: Supplementary Table 7 [file mmc7.docx]

*Supplementary Table 7. Ninety-Day Postoperative Outcomes Following Primary Total Shoulder Arthroplasty Comparing Aspirin 81 mg and Aspirin 325 mg*

| Outcome | ASA 81 mg (n = 14,972) | ASA 325 mg (n = 14,972) | RR [95% CI] | P value |
| --- | --- | --- | --- | --- |
| Readmission | 0.6% | 0.7% | 0.835 [0.630, 1.108] | 0.211 |
| ED Visit | 3.4% | 3.3% | 1.036 [0.883, 1.216] | 0.661 |
| PE | 0.4% | 0.3% | 1.060 [0.715, 1.572] | 0.770 |
| DVT | 0.4% | 0.6% | 0.720 [0.520, 0.998] | 0.047 |
| Myocardial infarction | 0.6% | 0.5% | 1.117 [0.811, 1.537] | 0.498 |
| SSI | 0.3% | 0.1% | 2.251 [1.330, 3.811] | **0.002** |
| PJI | 0.9% | 1.0% | 0.933 [0.734, 1.186] | 0.573 |
| Revision Arthroplasty | 1.0% | 0.8% | 1.285 [1.006, 1.643] | **0.044** |
