# Supplementary material for: Pre-operative antiplatelet therapy is associated with increased risk of periprosthetic joint infection following total shoulder arthroplasty
Source: J Shoulder Elb Arthroplast. 2026 Mar 3;10(1-2):100010. doi: 10.1016/j.jsea.2026.100010 (PMC13103263; doi:10.1016/j.jsea.2026.100010)
Supplement: Supplementary Table 8 [file mmc8.docx]

*Supplementary Table 8. Six-Months Postoperative Outcomes Following Primary Total Shoulder Arthroplasty Comparing Aspirin 81 mg and Aspirin 325 mg*

| Outcome | ASA 81 mg (n = 14,972) | ASA 325 mg (n = 14,972) | RR [95% CI] | P value |
| --- | --- | --- | --- | --- |
| Readmission | 0.7% | 0.9% | 0.771 [0.594, 1.001] | 0.050 |
| ED Visit | 5.3% | 5.1% | 1.030 [0.907, 1.170] | 0.650 |
| PE | 0.5% | 0.5% | 1.012 [0.730, 1.403] | 0.943 |
| DVT | 0.7% | 0.9% | 0.718 [0.554, 0.931] | **0.012** |
| MI | 0.8% | 0.8% | 1.031 [0.795, 1.337] | 0.820 |
| SSI | 0.4% | 0.2% | 2.208 [1.425, 3.422] | **<0.001** |
| PJI | 1.2% | 1.2% | 0.982 [0.797, 1.210] | 0.864 |
| Revision Arthroplasty | 1.6% | 1.2% | 1.332 [1.091, 1.625] | **0.005** |
