# Supplementary material for: Pre-operative antiplatelet therapy is associated with increased risk of periprosthetic joint infection following total shoulder arthroplasty
Source: J Shoulder Elb Arthroplast. 2026 Mar 3;10(1-2):100010. doi: 10.1016/j.jsea.2026.100010 (PMC13103263; doi:10.1016/j.jsea.2026.100010)
Supplement: Supplementary Table 9 [file mmc9.docx]

*Supplementary Table 9. One-Year Postoperative Outcomes Following Primary Total Shoulder Arthroplasty Comparing Aspirin 81 mg and Aspirin 325 mg*

| Outcome | ASA 81 mg (n = 14,972) | ASA 325 mg (n = 14,972) | RR [95% CI] | P value |
| --- | --- | --- | --- | --- |
| Readmission | 0.9% | 1.2% | 0.764 [0.608, 0.961] | **0.021** |
| ED Visit | 8.7% | 8.5% | 1.020 [0.926, 1.124] | 0.687 |
| PE | 0.8% | 0.8% | 1.108 [0.856, 1.434] | 0.436 |
| DVT | 1.1% | 1.3% | 0.790 [0.639, 0.977] | **0.029** |
| MI | 1.5% | 1.3% | 1.116 [0.918, 1.358] | 0.271 |
| SSI | 0.6% | 0.3% | 1.810 [1.268, 2.582] | **0.001** |
| PJI | 1.8% | 1.7% | 1.086 [0.913, 1.291] | 0.351 |
| Revision Arthroplasty | 2.3% | 1.8% | 1.279 [1.087, 1.505] | **0.003** |
